# A Siamese deep learning framework for efficient hardware Trojan detection using power side-channel data

**DOI:** 10.1038/s41598-024-62744-2

**Published:** 2024-06-06

**Authors:** Abdurrahman Nasr, Khalil Mohamed, Ayman Elshenawy, Mohamed Zaki

**Affiliations:** Faculty of Engineering, Systems and Computers Engineering Department, Al-Azhar University, Nasr City, Cairo, Egypt

**Keywords:** Hardware Trojan, Electromagnetic radiation, Side-channel analysis, Deep learning, Siamese neural network, Engineering, Mathematics and computing

## Abstract

Hardware Trojans (HTs) are hidden threats embedded in the circuitry of integrated circuits (ICs), enabling unauthorized access, data theft, operational disruptions, or even physical harm. Detecting Hardware Trojans (HTD) is paramount for ensuring IC security. This paper introduces a novel Siamese neural network (SNN) framework for non-destructive HTD. The proposed framework can detect HTs by processing power side-channel signals without the need for a golden model of the IC. To obtain the best results, different neural network models such as Convolutional Neural Network (CNN), Gated Recurrent Unit (GRU), and Long Short-Term Memory (LSTM) are integrated individually with SNN. These models are trained on the extracted features from the Trojan Power & EM Side-Channel dataset. The results show that the Siamese LSTM model achieved the highest accuracy of 86.78%, followed by the Siamese GRU model with 83.59% accuracy and the Siamese CNN model with 73.54% accuracy. The comparison shows that of the proposed Siamese LSTM is a promising new approach for HTD and outperform the state-of-the-art methods.

## Introduction

ICs go through a multi-step journey from defining their purpose (specification) to final product (assembly & packaging). IC’s designers prefer to resort to outsourcing as an efficient means of cost-effectiveness and process timesaving^1^. Hardware Trojan (HT) represents a malevolent alteration of an integrated circuit's circuitry, typically introduced either during its design phase through third-party involvement or during outsourcing. These illicit modifications are programmed to execute unauthorized functions when specific trigger conditions are met. Activating these triggers during testing phases is often challenging. HTs can be stealthy inserted at the design or fabrication stages and remain dormant until invoked. Their malicious consequences encompass a range of actions, including the leakage of sensitive data, the disabling of crucial systems, and the potential for catastrophic ramifications, especially when deployed in vital infrastructure sectors such as defense, communications, and energy^2^.

**Figure 1:**
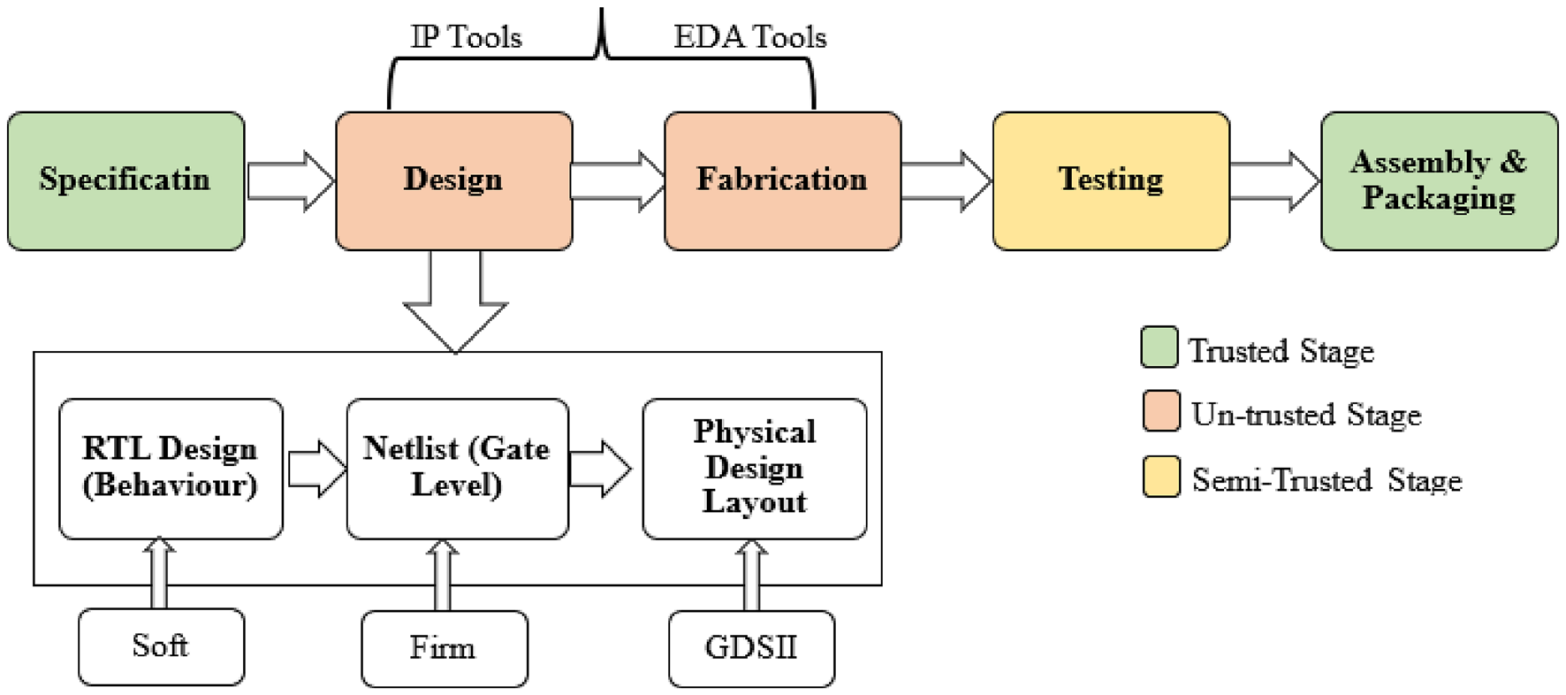
Stages in the IC production.

As shown in Fig. 1, the phases of the IC industry can be categorized according to the probability of exposure to the HT injection process as the following: (i) trusted stages, where HTs cannot inserted by attackers, (ii) untrusted stages, where HTs can easily inserted by Intellectual Property (IP) tools or Electronic Design Automation (EDA) tools, and (iii) semi-trusted stages: where there was a trouble for attacker to insert HTs, but it was possible to be inserted^3^.

HT consists of two main parts (i) a trigger circuit that monitors hardware signals for a specific event to activate the payload. (ii) The payload carries out the malicious behavior^4^. HTs are categorized into analog based and digital based on the trigger conditions. Digital HTs can be divided into combinational and sequential types^5^.

Sequential HTs are related to the main clocks of the original circuit^6^.

To solve the security issues caused by HTs, researchers have developed several Hardware Trojan Detection (HTD) techniques that can be applied with the aim of designing a secure IC that is free of HTs^7^. One way is to use secure design practices, such as secure IC design methodologies and secure supply chain management. Another way is to use hardware security testing techniques, such as functional testing, fault injection testing, and side-channel analysis. HTD can be categorized as follows:

i. Imaging techniques: The chip is delayered, figuring the die, the image of the circuit is engineering reversed, and conducting element-by-element comparisons. In this technique, a certain test record is used activate the HT, the tested device response is captured, and a HT is detected^8^. The main disadvantages of these techniques are they are destructive, non-practical, consume a large time, high cost, are not able to detect infected third-party Ips, and the complete assurance of identifying all design-stage HTs may not be achievable^9^.
ii. Side-channel analysis (SCA) technique: can be used to extract information from a system by observing its physical side-channels such as Power Consumption (PC), timing, Electromagnetic Radiation (EMR), temperature, path-delay analysis, power supply transient signals, leakage currents. These parameters are compared with the expected values to detect the presence of unwanted structure^10^. HT’s can introduce minor changes in the chip operation, PC and EM pattern based on the chip logic function. HT’s can alter this pattern by adding/modifying the chip function, which lead to a slight change in the chip PC. Similarly, the chip EM’s depend on its internal switching activity. HT’s can cause a slight change in these emissions due to localized changes in current density or altered timing characteristics.

SCA detects the HT by measuring the side-channel emission and compare it with the acquired one from a golden chip to create a reference model of expected side-channel values.

The main limitation of these methods is the need for a trusted golden chip for comparison which is expensive and too complex for exhaustive verification, especially in large designs, and may not be practical in real-world applications^11^. Also, the changes in chip PC and EM are often very small and require complex signal processing techniques, Additionally, factors such as manufacturing variations and environmental noise can further mask these changes, making HTD a complex task.

Traditional HTD techniques have several limitations such as it may reject a legitimate IC due to the false positive rate or deployment of a faulty IC due to a low detection rate, it is very expensive to implement and slow down the execution time of the IC functionality which makes them impractical in real-time applications. Machine Learning (ML) and Deep Learning (DL) can be used to improve the accuracy and efficiency of HTD. It can be used to learn the features that are characteristic of HT. DL techniques can be used to learn more complex features that are characteristic of HT, which helps to improve the detection rate of HTD techniques^12^.

Recently, various ML-based methods have been proposed to improve HTD techniques, such as K-Nearest Neighbors (KNN), naive Bayes, K-means, support vector machines (SVM), and neural networks^13,14^. By updating the HT database with ML, new types of trojans can be detected using the updated classifiers.

In this paper, a novel non-destructive Siamese based neural network framework for HTD in ICs is proposed and implemented. The proposed framework is free of complexities, and it is gust, a base line. It exploits similarity/dissimilarity learning to achieve self-refence characteristics, consequently, it is golden chip free and unaffected by any variations due to processing noise. Three types of neural networks are integrated with the SNN to construct the following models (i) Siamese Convolutional Neural Network (Siamese CNN), (ii) Siamese Gated Recurrent Unit (Siamese GRU), and (iii) Siamese Long Short-Term Memory (Siamese LSTM). These different models are trained and tested on the Trojan Power & EM Side-Channel dataset. A dataset of ICs that have been labelled as either malicious or benign. The obtained results are evaluated for all models and a comparison is performed between the performance of the three models and with the state-of-the-art methods. The main contribution of this paper is:

i. A novel non-destructive golden chip free Siamese HTD framework is presented to overcome the limitations of the traditional HTD techniques. Since it exploits only the electromagnetic radiations of the IC, rather than IC circuit diagram which eliminates the need for the golden chip.
ii. The proposed technique is implemented using different architectures CNN, GRU, and LSTM providing an average accuracy of 86.78%, 83.59%, and 73.54% respectively. The obtained results indicate that Siamese LSTM outperforms other architectures since it offers unmatched adaptability and scalability to new HTs, thus providing a robust solution to the dynamic challenge of HTD.
iii. The proposed framework behaviour is compared with the state-of-the-art HTD methods. The results shows that HTD using Siamese LSTM achieves high accuracy in detecting HTs compared with other traditional HTD methods, which can be expensive to implement due to its dependency on the golden chip.

The paper is organized as follows: Section two discusses “Related work”. Section “Threat model for SNN-based HT” describes the threat model for the proposed architecture. Section “The proposed HTD model” presents the proposed method for HTD using Siamese neural networks. Section “Implementation” describes the implementation of the proposed framework. Section six lists the “Experimental results”. Section “Performance analysis” analyzes the performance of the proposed framework. Section “Comparative study” compares the proposed algorithm to other algorithms. Section “Conclusion” concludes the paper.

## Related work

HTD methods can be classified into both traditional methods such as side-channel-based, and power-based side-channel analysis techniques, and AI-based techniques such as using ML and DL-based techniques.

### Traditional HTD techniques

Yuanwen et al.^11^ proposed a framework that are used in test generation to improve HTD sensitivity in analyzing side-channel. It involves increasing switching activity in an unknown HT statistically to enlarge the HT effect with the existence of huge process variations.

In^15^, the power profiles of trojan-free structures are used computer the temperature and power statistics of the IC. In^16^ the emission images of the IC are generated based on a trojan-free layout of this IC. In^17^, the authors indicates that power-based side-channel analysis may face potential vulnerabilities to Trojans that manipulate power control^17^. In^18^, a trojan-free structure of the chip is used to derive a golden model for that chip, which faces a problem to be implemented in practice due uncertainty about the chip is free of HTs. Balasch et al.^19^ propose a novel HTD technique based on fingerprinting the EM emissions of ICs to identify the HTs injected in different location in FPGA. In^20,21^, the authors discuss how HTs, and noise sources are detected based on EMR emitted from chips. They propose a specific noise model and study the effect of the noise types on the proposed model. Also, they discuss the link between the HT and the noise type, along with the possible limitations of EMR-based methods.

The most significant limitations of traditional HTD techniques are: (i) they are often not scalable to large ICs. (ii) They can be easily evaded by sophisticated attackers. (iii) They can be expensive and time-consuming to implement, and they often require the use of specialized hardware or software^3^.

### HTD based on ML

ML and DL are being increasingly used for HTD since they are able identify the complicated features from the IC's circuitry. In^22^, Nasr et al. propose a method for HTD in ICs, they implement HAAR algorithm to extract the required features and classify the possible HTs using Adaboost cascaded classifier. However, this approach has limitations, as it requires substantial human input and domain knowledge to extract features accurately, making it challenging to detect features from other domains. Additionally, it is not robust against image variations. Chen et al.^23^, present a ML-based approach for HTD in IoT chips. It involves feature extraction from the net and utilizes a scoring mechanism, XGBoost, to eliminate irrelevant features.

In^24^, Gowtham et al. apply a supervised ML approach and the random forest algorithm for HTD. The proposed approach utilizes five features extracted from gate-level netlists for each circuit net. In^25^, Hasegawa et al. propose a method for HTD by extracting 11 features from gate-level netlists.

They train a multi-layer neural network model during IC design, achieving 100% TPR. They improve feature extraction by selecting 11 effective features out of 51, leading to improved classification performance for all circuit nets.

In^26^, Lavanya et al. present a multi-parametric assessment model for detecting HTs using ensemble learning. This model utilizes delay, PC, and resource utilization profiles extracted from AES-256 circuits. Resampling techniques are applied to address data imbalance, and feature selection is performed using effective predictor, cross-correlation, and principal component analysis. The selected features are trained by an ensemble learning model comprising 11 base classifiers. The obtained results show that the proposed model provides a path-level trojan detection with high accuracy, outperforming existing approaches.

### HTD based on DL

In^27^ K. Reshma et al. propose a DL-based HTD using an autoencoder-based neural network to reduce features. The proposed model employs k-means clustering for classification and selects specific features, such as controllability and transition probability for analysis. In^28^, an HTD technique that employs deep-stacked autoencoders is proposed. The circuit features are extracted and entered to the DL model. The obtained results indicate that this method outperforms existing approaches, achieving a TNR of 95% and TPR of 75%. In^29^, S. Sankaran et al. present a DL-based HTD in IC chips. They inject trojans at the circuit level and collect PC data during normal and compromised operations. This approach includes neural networks and auto-encoders, to profile the normal behavior and identify anomalous PC patterns as potential signs of trojan activity.

In^30^, Sharma et al. present an HTD technique using Deep CNN (DCNN) that automatically extracts robust features from IC layout images. A new stopping condition method and metrics are introduced to prevent over-training of the model. The technique incorporates noisy images and applies data augmentation and regularization to address process variations and fabrication noise. It achieves 99% and 97.4% accuracy on Trust-Hub and synthetic ISCAS datasets, respectively. In^31^, Faezi et al. introduce a novel neural network for HTD (i.e., HTNet) in run time with no need for a trojan-free chip. They construct A library of HTs; electromagnetic (EM) and power side-channel signals are collected for each case. HTNet is trained in this library to learn the best discriminative features. During the testing phase, HTNet is fine-tuned to learn the behavior of the test chip. HTNet is used in conjunction with an anomaly detection algorithm to monitor the side-channel signals emitted from the chip and report malicious behaviors related to a triggered HT.

In^32^, Yasaei et al. propose a HTD method for both gate-level netlists and RTL. It uses Graph Neural Networks (GNNs) to predict the circuit behavior using a Data Flow Graph (DFG) representation of IC design. Fredin et al.^33^, suggest using gate-level netlists to detect HTs. The netlists are used to create datasets with extracted features for DL models. These models use the features to identify trojan characteristics and classify nets as either trojan-free or trojan-infected. A gate-level netlist-based system for HTD is presented in^34^. The system uses LSTM and CNN models to optimize training parameters. The system achieves high detection rates for both combinational HTD and sequential HTD. In^35^, Pan et al. propose a novel approach for HTD at the gate level using graph learning techniques with no need for a golden model and can be seamlessly integrated into the IC design flow. It utilizes a unionid GNN to combine information from different sides of the directed graph, achieving high detection performance. The experimental results demonstrate an average recall of 93.4. In^36^, Tong et al. propose a DL-based framework called SEM2GDS for HTD. It addresses the challenge of comparing SEM images with GDS images by transforming SEM images into GDS images with sharp corners. The proposed model achieves high accuracy for HTD, a higher F1 score, and a very low False Negative Rate (FNR) of 0.02.

In^37^, Sumarsono et al. propose a novel HTD technique using LSTM Auto Encoder (LSTM-AE). The LSTM-AE algorithm is demonstrated to effectively detect both always-on and condition-based HT. The activation characteristics of HT are categorized as always-on or triggered by specific conditions. The experimental results validate the viability of LSTM-AE for reliable HTD.

DL is effective for HTD. Underfitting and till some limitations to its use such as the need for large datasets, vulnerability to adversarial attacks, can be prone to overfitting or underfitting, and can be computationally expensive to train and deploy.

## Threat model for SNN-Based HT

In this section, the threat model for the proposed HTD using Siamese deep learning is outlined. The System threat mode is assessed to discover imminent threats in the pipeline of Siamese network. For example, a threat could be exploited to scramble the similarity learning process, parameter manipulation, or data poisoning, resulting in disastrous detection results and false classification. The threat model and defenses described below provide a starting point for understanding and protecting against possible attacks over the SNN-based HTD Techniques.

- **Threat landscape**: An attacker inserts malicious parameters, manipulates network configurations, poisons data or crafts sophisticated input vector into the SNN pipeline, specifically at the intersection of the twin node before the output. This manipulation is designed to scramble the similarity learning process, resulting in inaccurate detection results.
- **Attacker profile**: The attacker can be anyone who has access to the SNN pipeline implementation, or target system deployment. This could include a disgruntled employee, a competitor, or a foreign government.
- **Target**: The target of the attack is the SNN itself. This exploitation can be inserted into any SNN that is used for HTD.
- **Impact**: The SNN will no longer be able to accurately detect HTs. This could lead to a serious security breach, as attackers could use such attack vector to steal sensitive data, disrupt critical systems, or even cause physical damage.
- **Mitigation strategies**: To safeguard against these threats, we propose a multi-layered defense approach:
- ***Secure design practices***: Implement secure coding principles and robust network architectures to make tampering or exploitation more difficult.
- ***Data sanitization and validation***: Rigorously clean and validate training data to prevent data poisoning attempts.
- ***Adversarial training***: Expose the SNN to diverse and adversarial training samples to enhance its resilience against targeted attacks.
- ***Continuous monitoring***: Employ anomaly detection techniques and security monitoring tools to identify suspicious activity within the SNN and surrounding environment.
- ***Regular security assessments***: Implement ongoing evaluations for vulnerabilities and execute penetration tests to uncover and mitigate new threats.

## The proposed HTD model

This section outlines the approach selected for the HTD using the SNN structure. The methodology is rooted in the analysis of power and EM side-channel signals, aimed at discerning the presence of trojans within ICs. The SNN architecture employs various neural network layers, including LSTM, GRU, and CNN. The framework is executed within the Google Colab environment, harnessing the computational efficiency of the GPU processor.

At its core, the methodology centers on the SNN framework. SNNs are a fitting choice for HTD due to their unique architecture designed to capture complex patterns within data. By training on pairs of authentic and potentially compromised instances, SNNs naturally learn to distinguish subtle irregularities that indicate the presence of trojans. Each input layer of the SNN is optimized to interpret the intricacies of power and EM signals. SNN is constructed by combining recurrent, dense, and output layers.

The primary goal of the methodology is binary classification, aimed at distinguishing input vectors associated with trojan-free ICs from those indicating trojan presence. These input vectors, characterized by their univariate nature, encapsulate the nuances of power and EM signals, expressed as an array of 2500 components.

**Figure 2:**
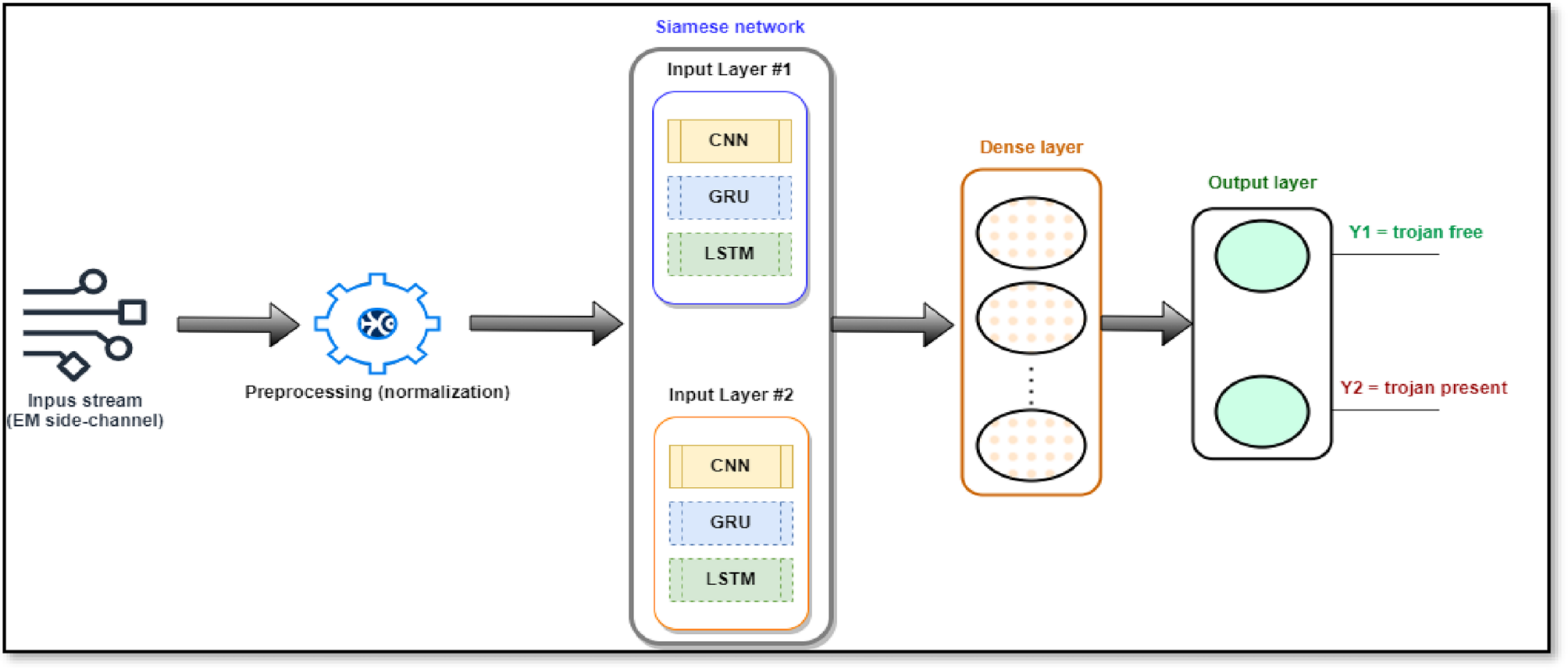
The proposed SNN architecture (the dashed line indicates that only one network is used during the experiment), in this figure the active network is the Siamese CNN.

Within the SNN architecture, Fig. 2, input vectors traverse parallel pathways, undergoing feature extraction and encoding via distinct neural network layers. This process captures the subtle dependencies and patterns inherent in the data, producing refined representations that enhance the network's trojan detection capability.

The methodology operates through two fundamental phases: (i) the training phase and (ii) the testing phase. During training, the SNN network learns iteratively from labelled data. This phase aids the network in understanding the distinguishing characteristics between trojan-free and trojan-laden instances. The testing phase involves a representative subset of data randomly selected from the IEEE Dataport "Hardware Trojan Power & Em Side-Channel" dataset^38^. This phase assesses the model's ability to accurately classify ICs, offering insights into the methodology's effectiveness.

The design of the SNN architecture within this research is underpinned by a deliberate emphasis on simplicity and lightweight characteristics of network design, yielding a model tailored for prompt and real-time decision-making response. Recently, researchers advocated for the development of lightweight models^39^ that can operate seamlessly in resource-constrained environments, such as embedded systems and edge devices.

This pursuit aligns with the demands of industries seeking to integrate intelligent functionalities into diverse applications without incurring substantial computational overhead. By adopting this design strategy, the proposed SNN architecture navigates the intricate landscape of HTD with an efficiency that accommodates the requirement for swift and real-time responses, offering a promising avenue for the deployment of trojan mitigation strategies in dynamic operational contexts.

In contrast to the typical application of SNN networks on 2D image inputs, this research introduces a novel utilization of the SNN architecture tailored for a distinctive context. While SNN networks have traditionally excelled in image similarity tasks, our approach transcends this conventional paradigm by harnessing the architecture's power for HTD using 1D power side-channel signals.

This innovative adaptation aligns with a broader trend observed in contemporary research and discussions across various academic and industrial platforms^40^. Notably, emerging studies have explored the versatility of neural network architectures beyond their original domains, showcasing their efficacy in novel applications, such as time-series data analysis and single-dimensional signal processing. The pioneering use of the SNN architecture to inspect 1D power side-channel signals underscores the adaptability and potential of DL techniques to transcend domain boundaries and address intricate challenges in hardware security.

The experimental setup embraces diversity by employing distinct neural network layers for input analysis. The LSTM, GRU, and CNN-based Siamese configurations facilitate a comprehensive exploration of model performance, providing insights into the most suitable architecture for HTD detection.

## Implementation

**Figure 3:**
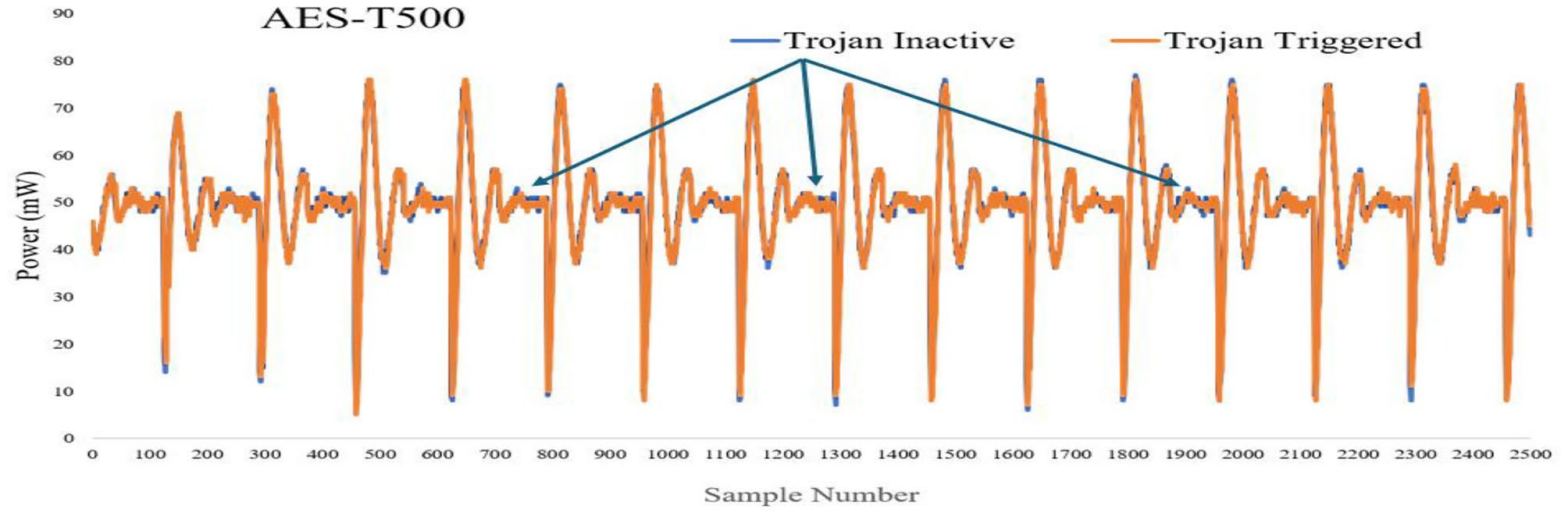
Power series trojan inactive/ trojan triggered.

The focal point of this section revolves around HTD utilizing the "Hardware Trojan Power & EM Side-Channel" dataset obtained from IEEE DataPort^41^. The IEEE dataset is a comprehensive collection of single-dimensional time series readings for power and EM side-channel signals, Fig. 3. Comprising authentic and potentially trojan-affected ICs, this dataset offers a representative sample of the hardware landscape. Each instance within the dataset is a vector of 2,500 components, capturing intricate variations in power and EM characteristics that could potentially unveil the presence of HTs.

The dataset collection condition depends on several factors including physical parameters, trojan type, IC input vector, and chip temperature.

This study was conducted within the Google Colab environment, utilizing the Python programming language. The computational power of the T4 GPU processing unit was harnessed to expedite the training and optimization of the DL models. The entire model is optimized by making use of the Adam optimizer to enhance performance and convergence during training. The models were trained for 5 epochs with a batch size of 128, ensuring a balance between convergence and computational efficiency. TensorFlow version 2.13 was employed, providing a stable foundation for model development.
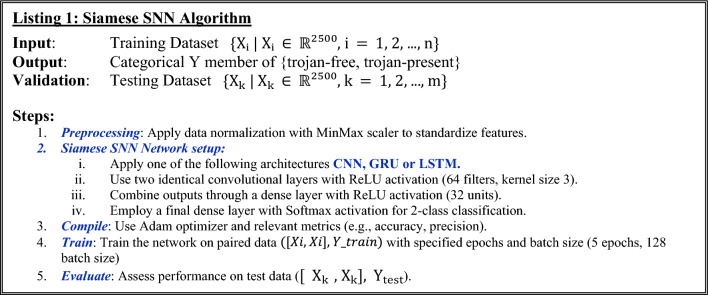


The comprehensive approach, encompassing the choice of network setup, dataset, hardware acceleration, epoch settings, batch size, and TensorFlow version, establishes a robust foundation for investigating and detecting HTs within ICs. This contribution advances the field of Trojan detection methodologies, paving the way for enhanced IC security.

The primary objective is to discern between ICs that are Trojan-present and those that are Trojan-absent, by employing the following three distinct DL methodologies:

### Siamese CNN for HTD

CNNs are a type of machine learning model that can learn to recognize patterns in data, such as images and text. CNNs work by breaking down the data into smaller pieces and then looking for patterns in those pieces. CNNs have been shown to be very effective at a wide range of tasks, such as image classification, object detection, and natural language processing.

For the Siamese CNN, the CNN architecture mirrors the configuration while introducing a kernel size of 3 for the convolutional layers and ReLU activation. To standardize the dataset, a min–max normalization process is employed as a preprocessing step. The data was randomly shuffled and partitioned into 70% for training and 30% for testing, with tenfold cross validation. Listing 1 shows a pseudo-implementation for the Siamese CNN architecture with CNN selected as the main architecture of the SNN.

### Siamese GRU for HTD

GRUs are a type of neural network that is good at learning patterns in sequential data, such as text or audio. GRUs do this by using gates to control which information is passed from one step in the sequence to the next. This allows GRUs to learn long-term dependencies in the data, which is important for many tasks such as machine translation and speech recognition.

For the GRU architectures, the configuration encompasses an initial layer of 64 input neurons utilizing the hyperbolic tangent (tanh) activation function, succeeded by a dense layer containing 32 input neurons with the rectified linear unit (ReLU) activation function. Subsequently, a final output layer with two neurons and a softmax activation function is employed to achieve classification. Listing 1 shows a pseudo-implementation for the Siamese GRU architecture with GRU is selected as the main architecture of the SNN.

### Siamese LSTM for HTD

LSTM is a type of recurrent neural network (RNN) that is designed to address the vanishing gradient problem found in traditional RNNs. The basic architecture of an LSTM consists of following three main components:

- **Input Gate**: It takes the current input and the previous hidden state as inputs and passes them through a sigmoid activation function. The output of the sigmoid function determines whether new information is stored in the LSTM cell or not.
- **Forget Gate**: The forget gate decides which information should be discarded from the memory cell based on the current input and the previous hidden state.
- **Output Gate**: it controls how much information from the memory cell should be used to compute the output at the current time step. The output of the output gate is the element-wise product of the sigmoid output and the tanh output, which gives the final output of the LSTM cell.

These gates work together to control the flow of information within the LSTM cell and regulate the memory storage and retrieval process. LSTM cells also have a cell state, which is responsible for storing and propagating information over time. The LSTM architecture's enables the network to remember relevant information over extended periods^42^.

**Figure 4:**
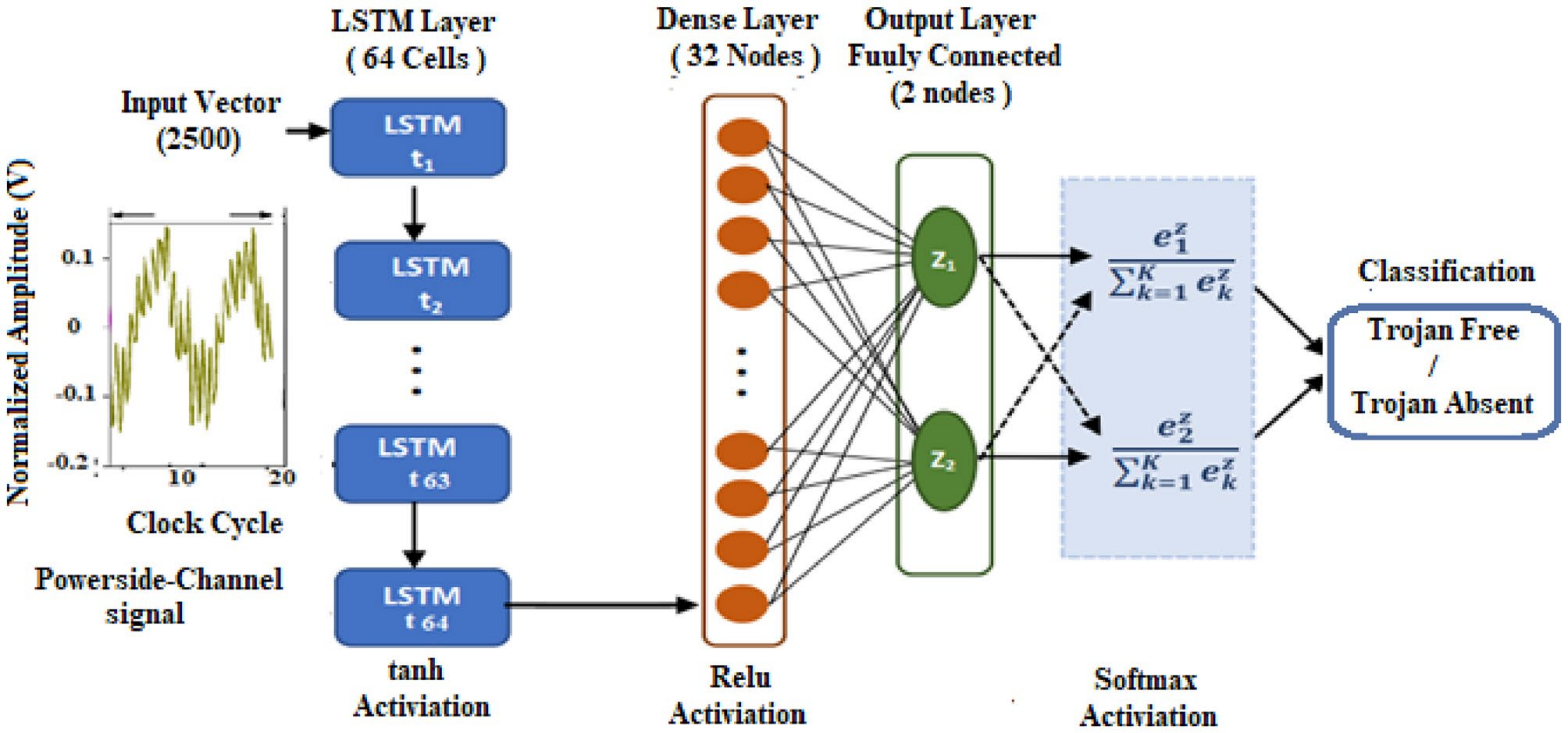
detailed architecture of SNN using LSTM**.**

The Siamese LSTM structure has a specific configuration. It begins with an input layer of 64 neurons, which receives the input data and applies the hyperbolic tangent (tanh) activation function. The tanh function scales the input values between -1 and 1, allowing the LSTM to capture non-linear relationships in the data. Figure 4 indicates the architecture of the implemented Siamese LSTM architecture.

Next is a dense layer of 32 neurons, which utilizes the rectified linear unit (ReLU) activation function. The ReLU function was considered for several reasons:

- **Computational efficiency**: Compared to other activation functions like sigmoid and tanh, ReLU requires fewer computations, leading to faster training and improved inference speed. This is crucial for real-time HTD applications.
- **Gradient flow**: ReLU avoids the vanishing gradient problem common in deeper networks, allowing gradients to flow more effectively during training and improving learning performance.
- **Sparsity**: ReLU outputs zero for negative inputs, creating sparsity in the network activations. This can help prevent overfitting and potentially improve generalization.

This introduces non-linearity and helps the LSTM model to learn complex patterns and features in the data.

The LSTM network concludes with a two-neuron output layer tasked with the classification process. This layer employs the softmax activation function to derive class probabilities, guaranteeing their collective sum equals one. Such a configuration enables the LSTM to offer decisive predictions, favoring the class deemed most probable. Listing 1 shows a pseudo-implementation for the SNN architecture with LSTM is selected as the main architecture of the SNN.

## Experimental results

The proposed SNN architecture is assessed using five statistical measures, namely:

i. **Accuracy**: The proportion of correctly predicted instances indicates how well the model classifies both trojan-free and trojan-present instances in the dataset.
ii. **FPR**: the proportion of instances that are incorrectly classified as positive (Trojan-present) out of all negative instances (Trojan-free).
iii. **TPR:** This metric reflects the ratio of accurately identified positive cases to the total number of true positives, highlighting the model's effectiveness in detecting the presence of Trojans.
iv. **F1 Score:** Represents the weighted average of precision and recall, serving as a comprehensive metric that balances the impact of false positives and false negatives on the model's performance.
v. **Error Rate:** Indicates the fraction of predictions the model incorrectly labels, shedding light on the precision of the model's predictive capabilities.

The SNN is trained on a dataset of ~ 60,000 samples of EM side-channel signals. Each sample represents a time series vector of 2500 sample points. The sample distribution of each class is almost equivalent. The network has been evaluated using the testing dataset that was highlighted in the implementation section.

The confusion matrices reported in Tables 1, 2 and 3 provide a detailed breakdown of the performance of three different SNN architectures GRU, CNN, and LSTM applied to HTD. These matrices offer valuable insights into each model's ability to correctly identify Trojan-present and Trojan-free instances, as well as the instances of false positives and false negatives.

As shown in table. 1, the Siamese GRU model shows robust ability to detect trojans with a relatively higher rate of False Positives (FP) of 5,441 instances compared to its performance on True Negatives (TN) of 4,514 instances. This suggests the model is somewhat more prone to erroneously flagging positive conditions as trojan-present.

**Table 1:** Siamese GRU confusion matrix.

Predicted values	Actual values	
	Positive	Negative
Positive	26,531	5441
Negative	4514	24,214

As shown in Table 2, the Siamese CNN model demonstrates a lower effectiveness in distinguishing between trojan-present and trojan-free instances compared to the GRU model, as indicated by higher counts of both FP (8,330) and FN (7,731). This suggests a balance issue in the model's sensitivity and specificity.

**Table 2:** Siamese CNN confusion matrix.

Predicted values	Actual values	
	Positive	Negative
Positive	23,314	8330
Negative	7731	21,325

As shown in Table 3, the Siamese LSTM model outperforms both the GRU and CNN models in terms of both sensitivity (lower FN) and specificity (lower FP). The reduced number of FP (4,219) and FN (3,801) suggests that the LSTM model is the most reliable HTD architecture that correctly classifying both trojan-present and trojan-free instances.

**Table 3:** Siamese LSTM confusion matrix.

Predicted values	Actual values	
	Positive	Negative
Positive	27,244	4219
Negative	3801	25,436

As shown in Table 4, Siamese LSTM and Siamese GRU achieved accuracy and F1 Score close to each other, while Siamese CNN achieved a lower accuracy and F1 Score, this suggests that Siamese LSTM and Siamese GRU are both effective for HTD using IEEE EM side-channel signal, while Siamese CNN is less effective.

**Table 4:** Evaluation metrics.

Network	Accuracy (%)	FPR (%)	TPR (%)	F1Score (%)	Error rate (%)	Precision (%)	Recall (%)
Siamese LSTM	86.8	14.2	87.7	87.1	13.2	86.6	87.8
Siamese GRU	83.6	18.3	85.4	84.2	16.4	83.0	85.5
Siamese CNN	73.5	28	75	74.3	26.5	73.7	75.1

For the Siamese GRU model, the Precision is approximately 83.0%, and the Recall is 85.5%. This suggests that while the model is relatively precise in its positive predictions, it also has a strong ability to capture most of the actual positive instances.

The Siamese CNN model shows a Precision of 73.7% and a Recall of 75.1%. These figures indicate that the CNN model is less precise in its positive predictions compared to the GRU model and slightly less effective at identifying all actual positives.

For the Siamese LSTM model, the Precision significantly increases to 86.6%, and the Recall is 87.8%. These results highlight the LSTM model's superior performance in both accurately predicting positive instances and effectively capturing nearly all actual positives.

The proposed SNN HTD framework was validated through a series of steps, considering the details of the dataset used for training and testing, as well as the obtained results and the ROC and AUC values.

Firstly, a dataset consisting of power and EM side-channel signals from HT benchmarks derived from Trust Hub was employed. The benchmarks targeted an encryption core circuit utilizing AES 128-bit encryption, where the key played a crucial role in maintaining the security of the encryption process.

The SNN HTD framework is implemented using GRU, CNN and LSTM models. These models were trained using the dataset to learn the patterns and characteristics of normal IC behavior, as well as to identify anomalies associated with Hardware Trojans.

**Figure 5:**
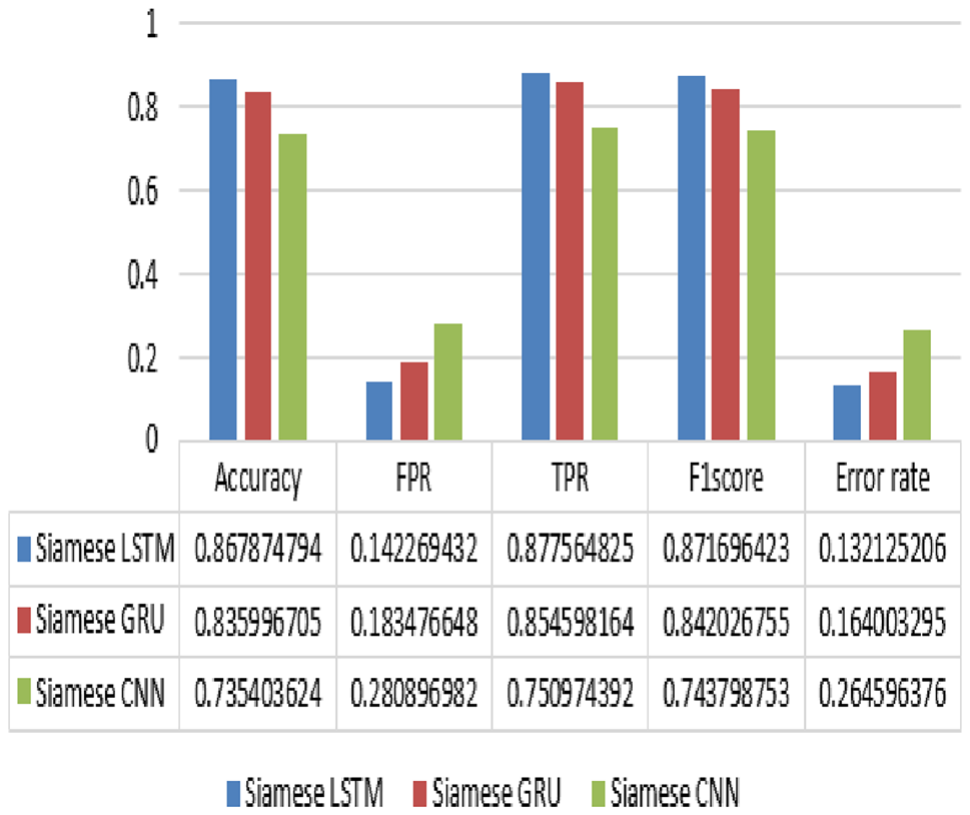
The performance evaluation of the proposed model.

In the training phase, the models were evaluated using various performance metrics as depicted in Fig. 5. The Siamese LSTM achieved an accuracy of 0.87, a False Positive Rate (FPR) of 0.14, a True Positive Rate (TPR) of 0.88, an F1 score of 0.87, and an error rate of 0.13. The Siamese GRU model achieved an accuracy of 0.86, an FPR of 0.18, a TPR of 0.85, an F1 score of 0.84, and an error rate of 0.16. Lastly, the Siamese CNN model achieved an accuracy of 0.74, an FPR of 0.28, a TPR of 0.75, an F1 score of 0.74, and an error rate of 0.26.

The uniqueness of the proposed methodology employing the Siamese network manifests in various facets, distinguishing it from prevalent HT localization techniques. Primarily, it adopts a self-reference approach, eliminating the reliance on golden chip reference designs to uncover HTs.

Moreover, it operates as a fully automated real-time solution, alleviating the necessity for labor-intensive manual feature extraction by experts—an approach prone to errors and time inefficiency. Additionally, this methodology yields commendable accuracy in HT identification, relieving designers of the Labor to inspect potential anomalous regions. Furthermore, its scalability and simple design a notable strength, permitting its application to expansive designs such as embedded systems and edge devices.

## Performance analysis

**Figure 6:**
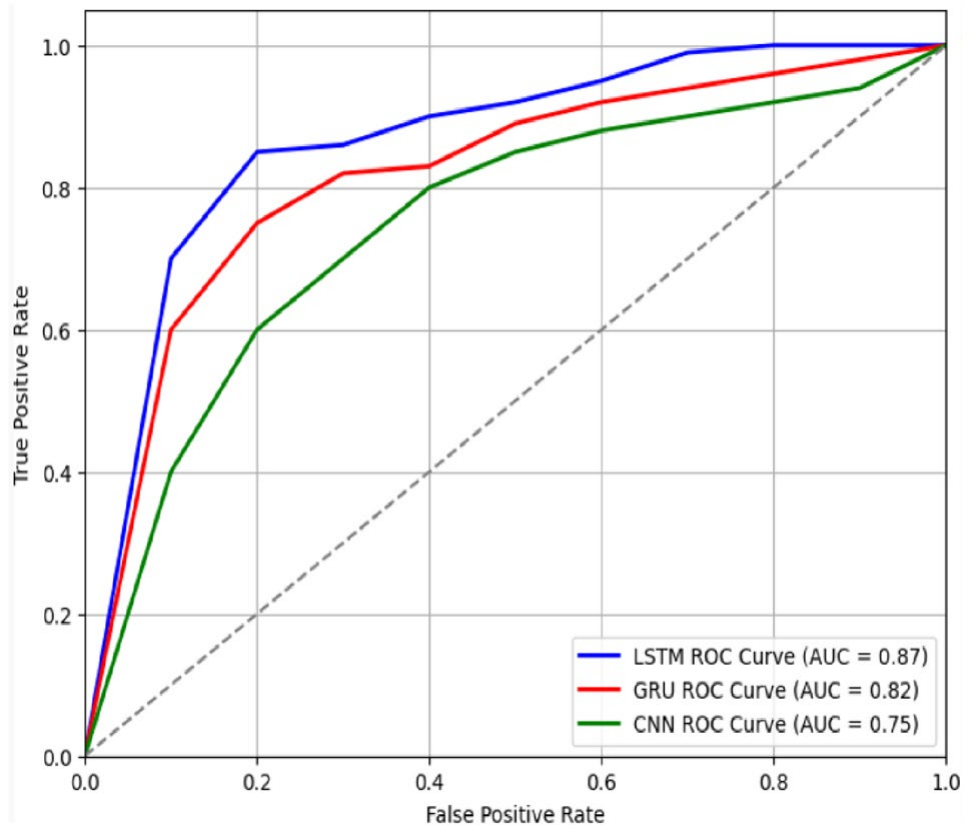
ROC and AUC of the proposed model.

The performance evaluation of the proposed model is shown in Fig. 5, and The ROC curve and AUC are summarized in Fig. 6. The Receiver Operating Characteristic (ROC) curve provides a graphical representation of the trade-off between the TPR (sensitivity) and the FPR (1 − specificity) for different classification thresholds. On the other hand, the AUC is a quantitative measure of the overall performance of a classifier, calculated as the area under the ROC curve. In the context of our study, we analyzed the ROC curves and corresponding AUC values for three different models as indicated in Fig. 6. These AUC values indicate the discriminatory power and performance of each model. The higher the AUC, the better the model's ability to distinguish between positive and negative samples. Based on the AUC values, the LSTM model exhibited the highest discrimination power, followed by the GRU model, while the CNN model showed a slightly lower performance. The ROC and AUC metrics provide valuable insights into the performance of the Siamese model, assisting in the selection and comparison of different classifiers for HTD.

There are a few possible reasons for this difference in performance. First, LSTM and GRU are both recurrent neural networks, which means that they can learn long-term dependencies in the data. This is important for HTD, as the EM side-channel signal can vary over time as the circuit executes different instructions. CNNs, on the other hand, are not as good at learning long-term dependencies. Second, LSTM and GRU have more parameters than CNNs. This means that they can learn more complex patterns in the data. CNNs, on the other hand, are more limited in their ability to learn complex patterns.

Finally, LSTM and GRU are more computationally expensive than CNNs. This means that they can be slower to train and to evaluate. However, the increased accuracy of LSTM and GRU may be worth the increased computational cost in some cases.

In summary, our results suggest that Siamese LSTM and Siamese GRU are both effective for HTD using IEEE EM side-channel signals. Siamese CNN is less effective, but it may be a better choice in cases where computational resources are limited.

## Comparative study

In this section, the accuracy of HTD using Siamese LSTM structure is quantitatively compared with the state-of-the-art HTD methods that applied on the same dataset (Trojan Power & EM Side-Channel dataset). Since the accuracy is the only common metrics used in these papers.

As shown in Table 5, the average accuracy of different methods for HTD are presented. The results show that the proposed Siamese LSTM network is the best performing method, with an average accuracy of 86.78%. This is followed by the Multilayer perceptron (MLP) network, with an average accuracy of 86.58%. The CNN network and the Hierarchical temporal memory (HTM) network also perform well, with average accuracies of 84.00% and 85.60%, respectively. The CNN with zero-shot learning (CNN-Z) and MLP with zero-shot learning (MLP-Z) networks perform worse than the other methods, with average accuracies of 64.86% and 63.33%, respectively. Although the improvement occurred by the proposed Siamese HTD may appear marginal (which is 0.20% over MLP), it's crucial to emphasize the significance of the model's ability to detect subtle and complex trojans, thereby reducing potential security risks. Moreover, a small subset of HT benchmark was utilized for training and testing. Overall, the results suggest that the Siamese LSTM network is a promising method for HTD.

**Table 5:** Comparison of Siamese HTD using LSTM with other methods.

Paper	Method	Average Accuracy
12	CNN	84.00
	MLP	86.58
	CNN-Z	64.86
	MLP-Z	63.33
	OC-SVM	70.25
	HTM	85.60
Ours	Siamese LSTM (Ours)	86.78

## Conclusion

In this paper, A novel non-destructive golden chip-independent Siamese HTD framework is proposed and implemented marking a significant advancement in the field of HTD. Unlike conventional HTD methods that rely on direct analysis of the chip circuitry or require a comparison against a golden chip model, our approach harnesses EMR data from the chip, leveraging this unique side-channel to detect the presence of HTs. This methodology not only sidesteps the limitations associated with traditional circuit inspection and golden chip comparisons but also offers a more scalable and easily deployable solution for real-world applications. The proposed framework is implemented using different architectures of SNN, including Siamese GRU, Siamese CNN and Siamese LSTM. The empirical results obtained from these implementations clearly demonstrate the superior performance of our framework, particularly highlighting the Siamese LSTM model as the most effective in identifying HTs with high reliability. This outcome not only underscores the potential of Siamese LSTM over other architectures within our framework but also positions it favourably against existing state-of-the-art HTD methods.

The insights gained from this study highlight the untapped potential of deep learning in enhancing the resilience of hardware systems against malicious alterations, setting a new benchmark for the development of robust, efficient, and non-invasive hardware Trojan detection methodologies.

## Data Availability

This work used the Hardware Trojan Power & EM Side-channel Datatset, which is publicly available on IEEEDataPort (https://ieee-dataport.org/3599).
